# Trends in perinatal deaths from 2010 to 2013 in the Guatemalan Western Highlands

**DOI:** 10.1186/1742-4755-12-S2-S14

**Published:** 2015-06-08

**Authors:** Ana Garces, Elizabeth M Mcclure, K Michael Hambidge, Nancy F Krebs, Lester Figueroa, Marta Lidia Aguilar, Janet L Moore, Robert L Goldenberg

**Affiliations:** 1 Francisco Marroquin University, Guatemala City, Guatemala; 2 FANCAP, Guatemala City, Guatemala; 3 Research Triangle International, NC, USA; 4 University of Colorado, Denver, CO, USA; 5 Columbia University, New York, NY, USA

## Abstract

**Background:**

While progress has been made in reducing neonatal mortality in Guatemala, stillbirth and maternal mortality rates remain high, especially among the indigenous populations, which have among the highest adverse pregnancy-related mortality rates in Guatemala.

**Methods:**

We conducted a prospective study in the Western Highlands of Guatemala from 2010 through 2013, enrolling women during pregnancy with follow-up through 42-days postpartum. All pregnant women were identified and enrolled by study staff in the clusters in the Chimaltenango region for which we had 4 years of data. Enrolment usually occurred during the antenatal period; women were also visited following delivery and 42-days postpartum to collect outcomes. Measures of antenatal and delivery care were also obtained.

**Results:**

Approximately four thousand women were enrolled annually (3,869 in 2010 to 4,570 in 2013). The stillbirth rate decreased significantly, from 22.0 per 1000 births (95% CI 16.6, 29.0) in 2010 to 16.7 (95% CI 13.5, 20.6) in 2013 (p-value 0.0223). The perinatal mortality rate decreased from 43.9 per 1,000 births (95% CI 36.0, 53.6) to 31.6 (95% CI 27.2, 36.7) (p-value 0.0003). The 28-day neonatal mortality rate decreased from 28.9 per 1000 live births (95% CI 25.2, 33.2) to 21.7 (95% CI 17.5, 26.9), p-value 0.0004. The maternal mortality rate was 134 per 100,000 in 2010 vs. 113 per 100,000 in 2013. Over the same period, hospital birth rates increased from 30.0 to 50.3%.

**Conclusions:**

In a relatively short time period, significant improvements in neonatal, fetal and perinatal mortality were noted in an area of Guatemala with a history of poor pregnancy outcomes. These changes were temporally related to major increases in hospital-based delivery with skilled birth attendants, as well as improvements in the quality of delivery care, neonatal care, and prenatal care.

## Background

Each year, families in low and middle income (LMIC) countries face the death of approximately 4 million newborns, 3 million fetuses and 300,000 pregnant women (99% of those that occur worldwide) [1-4]. Millennium Development Goal 4 (MDG4), which called for a two-thirds reduction in child mortality by 2015, depended in large part on the reduction of neonatal deaths, which made up 37% of child mortality in 1990 and 42% in 2014 [1,5]. The main causes of neonatal mortality in LMIC are preterm birth, birth asphyxia and infections; one third of deaths occur during the first 24 hours of life [3,6-8]. There is evidence of accelerating declines in neonatal mortality from 2000 to 2010 in many LMIC; reasons for these decreases include the introduction of national policies to promote development and increased access to health care, rising income of families and improved maternal education [9].

Third trimester stillbirths are a relatively common adverse pregnancy outcome in LMIC that in many instances, are potentially preventable [10-12]. Up to 70% of these deaths occur in the the intra-partum period in LMIC [8,12]. The main causes of stillbirths are asphyxia due to obstructed labor, placental abruption, preclampsia or eclampsia and umbilical cord complications [7]. Despite their high frequency, stillbirths were not included in the MDGs or in the Countdown to 2015 and are often not considered when pregnancy outcomes are reported from LMIC [12,13]. Because of their common causality, interventions that reduce stillbirths frequently reduce maternal and neonatal mortality [12].

An estimated 60 million women give birth each year outside of health facilities, mainly at home [8]; fifty-two million of these births occur without the assistance of a skilled birth attendant, frequently by traditional birth attendants (TBAs) or a family member [14]. In an effort to improve pregnancy outcomes overall, the World Health Organization (WHO) recommends the use of skilled birth attendants for delivery, as TBA training for the reduction of stillbirths and neonatal deaths has not been proven to be effective [7]. TBAs in Guatemala, known as *comadronas*, have a large role as providers of reproductive health services, in addition to being community leaders [14]. Eighty five percent of TBAs in the Chimaltenango region report one month or less of formal training, 60% are illiterate and less than one fourth report using a stethoscope for heart rate auscultation [14].

Chimaltenango is one of 22 states in Guatemala. Located in the western highlands, it has a population of 595,000, out of which 75% of inhabitants are indigenous and 50% live in rural areas. The United Nations human development index (HDI) for the region is 0.679, slightly lower than the HDI for the country (0.702). The maternal, fetal and neonatal mortality rates in this state are substantially higher than in other states [15]. In addition, indigenous peoples face the highest poverty rates.

We sought to explore the changes that have occurred in maternal deaths, neonatal deaths, stillbirths, and perinatal deaths between 2010 and 2013 in the Chimaltenango region of the Guatemalan Western Highlands. We also explored which characteristics and changes in these characteristics in the mothers, infants, or health services were temporally related to the decreases in mortality. This in-depth study of a particular geographic allowed us to understand the trends, and, if improvements occurred, the factors related to those improvements. The Every Newborn Action Plan, established by the World Health Assembly in 2014, calls for neonatal mortality and stillbirth rates to fall to below 10 per 1000 births by 2030 [6,13]. The present analysis should contribute evidence for the attainment of these goals in Guatemala.

## Methods

The study was conducted as part of the Global Network Maternal and Newborn Health Registry (MNHR) study, a multi-country research study. The purpose of this registry is to quantify and analyze trends in pregnancy outcomes in defined low-resource geographic areas over time in order to provide population-based statistics on pregnancy outcomes. The MNHR study enrols all pregnant women in the cluster, a defined geographic area. These analyses focus on the MNHR study clusters in the Chimaltenango region from January 1 2010 through December 31, 2013 (the last available data), in order to evaluate trends over a sufficient time period.

**Figure 1:**
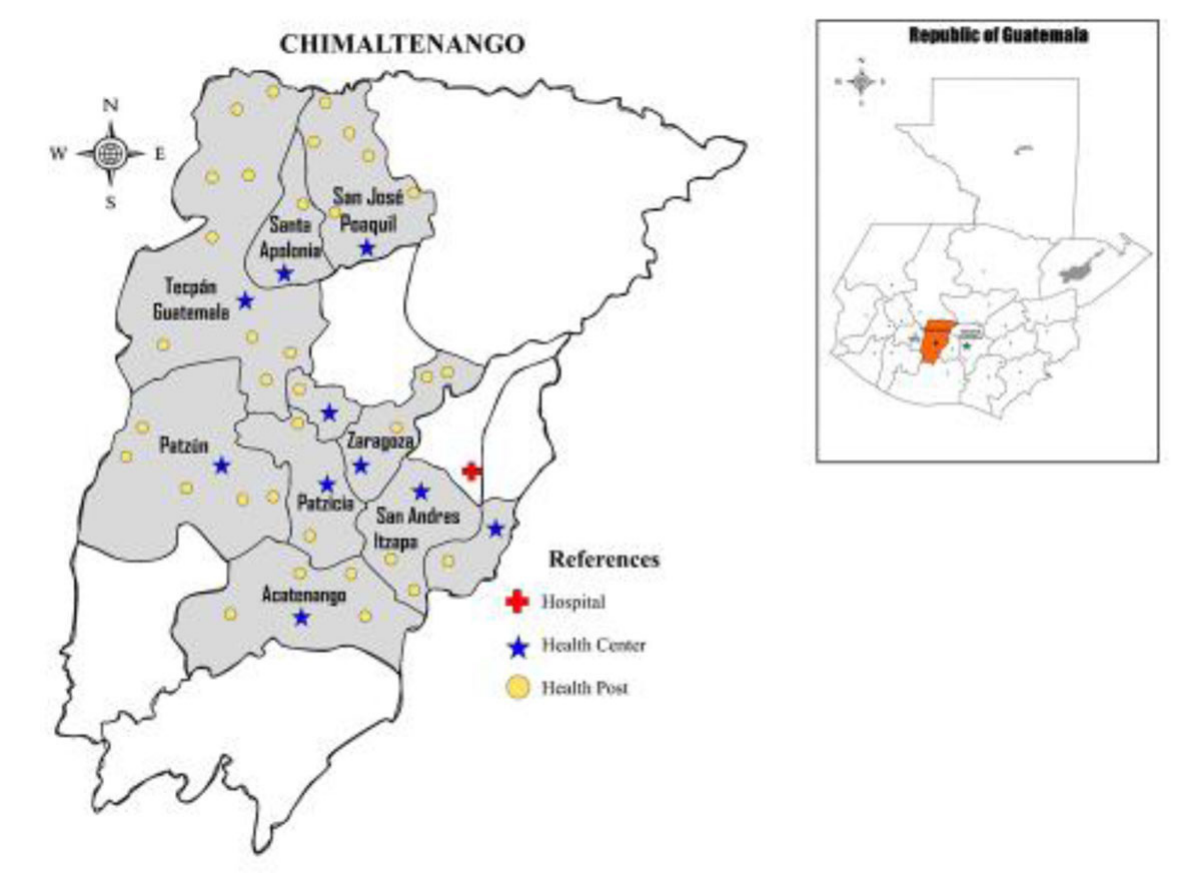
Map of Chimaltenango and Guatemala

The Chimaltenango study area expanded the number of clusters over time for the MNHR(Figure 1). However, for the purpose of this study, we restricted analyses to the subset of 10 clusters which were constant throughout the time period. Each of the clusters we analysed have one health center and between two to ten health posts, where ambulatory care is provided. There is only one referral hospital in the region. The referral system recommends that women be referred from the community level to health posts or health centers for ambulatory care and that all deliveries (especially of nulliparous women and any other with a complication), be referred to the Chimaltenango Hospital. While this option is available free of cost to any woman, to date, many prefer to have delivery at home, attended by a TBA.

To be informed of pregnancies in the communities, auxiliary nurses work together with the TBAs and the Ministry of Health services. The auxiliary nurses, who are full time study staff, visit women in their homes during pregnancy, following delivery (in the hospital or in the home, depending on where delivery occurred) and at six weeks post-partum in order to collect data on the health services and basic outcomes for each woman. All pregnancies, deliveries, stillbirths and deaths are checked against the data provided by local Ministry of Health and civil registry records. These data represent between 24 to 29% of all births in the department (state) of Chimaltenango for this time period.

Pregnancies were diagnosed mainly by self-referral of the mothers, by report of the TBAs, or by Ministry of Health records. All maternal deaths, including those in the first trimester, were included in the analysis. The gestational age was determined mainly by last menstrual period (LMP), as ultrasound gestational age determination was not widely available during this time period. Preterm birth was defined by LMP, report of the birth attendant or by using low birth weight as a proxy.

Stillbirths were defined as all fetal deaths at greater than 20 weeks gestation per 1000 births (live and stillbirths). Neonatal morality was defined as all neonatal deaths <28 days per 1000 live births. Finally, perinatal mortality was defined as all fetal deaths ≥20 weeks gestation plus early <7 day neonatal deaths per 1000 births. Maternal mortality was defined as all maternal deaths per 100,000 deliveries. We defined facility births as any delivery occurring within a health facility and community births as any delivery occurring at home or a TBA’s home.

Data were entered at a secured data computer at the research site located in Chimaltenango. Data were reviewed and edited on site, prior to transmission to the central data coordinating center (RTI International, US). Further edits were performed at the central data center, which were then resolved at the Guatemala data center. Data analyses included descriptive statistical analyses, to evaluate the rates, means and standard deviations. We used generalized estimating equations to account for correlation of outcomes within clusters to assure appropriately sized p-values. To evaluate changes in outcomes over time, we modelled year of delivery as a categorical value and tested for changes between years 2010 and 2013 with a simple difference contrast and also conducted trend tests. All data were analyzed using SAS v9.3 (Cary, NC).

### Ethics statement

The study was reviewed and approved by the ethics committee of Francisco Marroquin University, Guatemala as well as by the collaborating institutions at University of Colorado-Denver, Columbia University and RTI International, the data center. All women provided informed written consent for data collection prior to their study enrolment.

## Results

### Enrollment

Table 1 summarizes the annual enrolment in the MNHR in the clusters over the period, 2010 through 2013. Approximately four thousand women were enrolled each year (ranging from 3,909 women in 2010 to 4,661 in 2013) with delivery outcomes available for 99.1% (2010) to 97.5% (2012) of the women. During that period, a total of 16,702 fetuses/newborns were documented, with 6-week outcome available for 99.7% of all deliveries.

**Table 1:** Registry enrollment and follow-up by year in Guatemala, 2010-2013

	2010	2011	2012	2013	Total
Women enrolled, N	3,909	4,128	4,186	4,661	16,884

Women with deliveries, N (%)	3,875 (99.1)	4,096 (99.2)	4,083 (97.5)	4,585 (98.4)	16,639 (98.5)

Births, N	3,900	4,127	4,108	4,617	16,752

Excluded (< 1000g or miscarriage/mtp), N (%)	9 (0.2)	10 (0.2)	14 (0.3)	17 (0.4)	50 (0.3)

Births included, N	3,891	4,117	4,094	4,600	16,702

Six week neonatal outcome obtained, N (%)	3,870 (99.5)	4,106 (99.7)	4,091 (99.9)	4,584 (99.7)	16,651 (99.7)

### Trends in mortality rates

Table 2 displays the maternal, stillbirth, neonatal and perinatal mortality rates in the Chimaltenango region of the Western Highlands of Guatemala from 2010 to 2013. Over that period, the stillbirth rate decreased significantly from 22.0 per 1000 births (95% CI 16.6, 29,0) in 2010 to 16.7 (95% CI 13.5, 20.6) in 2013 (p-value 0.0223). Similarly the perinatal mortality rate decreased from 43.9 per 1,000 births (95% CI 36.0, 53.6) to 31.6 (95% CI 27.2, 36.7) (p-value 0.0003). Finally, the 28-day neonatal mortality rate decreased from 28.9/1000 live births (95% CI 25.2, 33.2) to 21.7 (95% CI 17.5, 26.9), p-value 0.0004. The maternal mortality rate per 100,000 was also lower (134 per 100,000 in 2010 vs. 113 per 100,000 in 2013), but the decrease was not statistically significant.

**Table 2:** Adjusted mortality rates by year, 2010 – 2013

	2010	2011	2012	2013	Reduction 2010 - 2013 (p-value*)
Women enrolled, N	3,909	4,128	4,186	4,661	

Stillbirth, Rate/1,000 births (95% CI)	22.0 (16.6, 29.0)	18.9 (13.3, 26.8)	16.1 (10.6, 24.6)	16.7 (13.5, 20.6)	0.0223

Perinatal mortality, Rate/1,000 births (95% CI)	43.9 (36.0, 53.6)	31.6 (27.2, 36.8)	27.9 (21.5, 36.2)	31.6 (27.2, 36.7)	0.0003

28-d Neonatal mortality, Rate/1,000 live births (95% CI)	28.9 (25.2, 33.2)	19.4 (16.2, 23.1)	17.3 (12.3, 24.3)	21.7 (17.5, 26.9)	0.0004

Maternal mortality, n/N (Rate/100,000, 95% CI)	134 (49, 366)	101 (40, 257)	199 (92, 429)	113 (38, 334)	0.7382

*P-value calculated for estimated reduction in adjusted rate 2010 vs. 2013; rates adjusted for cluster

In order to assess the factors potentially related to the improvements in mortality described above, we explored maternal characteristics, newborn characteristics, and quality of care overall.

### Maternal and newborn characteristics

Trends in certain maternal characteristics are shown in Table 3. Fifteen percent of mothers were less than 20 years of age, and 11% were more than 35 years of age; no substantive changes occurred in the maternal age distribution over the study time period. The proportion of women with no education significantly decreased from 24.2% to 17.3% and women with primary education or higher increased from 75.8% to 82.7% (p<0.05). There was a decrease in parity; the proportion of mothers with more than two births decreased from 37.9 to 33.8% and women who were primiparous increased significantly (p=0.02). The mean body mass index for women at antenatal care was approximately 27 kg/m^2^ and remained constant over the course of the study.

**Table 3:** Maternal characteristics by year in Guatemala

	2010	2011	2012	2013
Women with deliveries, N	3,869	4,088	4,073	4,570

Maternal age, N (%)				

< 20	556 (14.4)	622 (15.2)	647 (15.9)	703 (15.4)

20-35	2,858 (73.9)	3,015 (73.8)	2,990 (73.4)	3,406 (74.6)

> 35	451 (11.7)	448 (11.0)	436 (10.7)	459 (10.0)

Maternal education, N (%)				

No formal education	934 (24.2)	966 (23.6)	812 (19.9)	791 (17.3)

Primary	2,364 (61.2)	2,514 (61.5)	2,527 (62.0)	2,900 (63.5)

Secondary	506 (13.1)	579 (14.2)	701 (17.2)	841 (18.4)

University +	58 (1.5)	27 (0.7)	33 (0.8)	38 (0.8)

Parity, N (%)				

0	1,062 (27.4)	1,130 (27.6)	1,185 (29.1)	1,327 (29.0)

1-2	1,341 (34.7)	1,446 (35.4)	1,476 (36.2)	1,696 (37.1)

> 2	1,466 (37.9)	1,511 (37.0)	1,412 (34.7)	1,546 (33.8)

BMI at ANC, Mean (n, std)	27.0 (874, 3.9)	26.8 (906, 3.8)	26.7 (1960, 3.8)	26.8 (2885, 3.9)

A statistically significant increase in preterm births was reported over this time period (p<0.05), with a simultaneous increase in the proportion of babies with birth weights between 1500 and 2499 grams (also likely to be preterm births) (Table 4).

**Table 4:** Birth weight and gestational age by year in Guatemala

	2010	2011	2012	2013
Births, N	3,891	4,117	4,094	4,600

Birth weight, N (%)				

1000-1499g	15 (0.4)	25 (0.6)	17 (0.4)	42 (0.9)

1500-2499g	444 (11.5)	509 (12.4)	428 (10.5)	610 (13.3)

≥ 2500g	3,404 (88.1)	3,575 (87.0)	3,646 (89.1)	3,946 (85.8)

GA, N (%)				

Preterm	188 (5.1)	233 (5.9)	273 (6.7)	379 (8.3)

Term	3,504 (94.9)	3,695 (94.1)	3,795 (93.3)	4,187 (91.7)

Table 5 displays data related to antenatal care. The proportion of women with more than three antenatal care visits increased significantly from 79.1 to 93.3%, and the coverage of interventions provided improved overall. Access to vitamins and iron increased from 87.0% to 92.1% while the proportion of women tested for syphilis and HIV increased from 11.1 to 69.9% and 12.4 to 69.9% respectively. The tetanus toxoid vaccination decreased substantially from 87.8% to 60.6% over the study period (this apparent decrease was due to a change in the local Ministry of Health policy, in which women with a tetanus vaccination in the last ten years were not revaccinated). When we evaluated the trend, all changes were significant at p<0.05.

**Table 5:** Prenatal care in Guatemala’s Registry by year, 2010-2013

	2010	2011	2012	2013
Deliveries, N	3,869	4,088	4,073	4,570

ANC visits, N (%)	--	2,169	4,006	4,569

0	--	80 (3.7)	39 (1.0)	43 (0.9)

1-2	--	374 (17.2)	427 (10.7)	263 (5.8)

≥ 3	--	1,715 (79.1)	3,540 (88.4)	4,263 (93.3)

HIV testing received, N (%)	478 (12.4)	1,445 (35.7)	2,414 (59.9)	3,154 (69.9)

Vitamins/Iron, N (%)	3,359 (87.0)	3,559 (87.1)	3,671 (90.2)	4,205 (92.1)

Syphilis testing received, N (%)	427 (11.1)	1,300 (31.9)	2,405 (59.6)	3,154 (69.9)

				

Tetanus toxoid vaccine, N (%)	3,383 (87.8)	2,944 (72.3)	2,258 (55.5)	2,726 (60.6)

### Quality of care characteristics

During the 2010 – 2013 time period, changes also occurred in the characteristics of care during delivery (Table 6). The proportion of births attended by skilled health personnel increased from 33.1% to 55% while births attended by TBAs decreased from 66.6% to 44.4%. Births inside a hospital or clinic increased from 32.7% to 55.0% and the use of caesarean section increased from 13.3% to 24.3%. Both the use of maternal antibiotics and use of oxytocics significantly increased from 12.6% to 30.7%, and from 18.7% to 40.0%, respectively. Blood transfusion increased from 0.2% to 0.7%. Additionally, both the use of a clean razor to cut the umbilical cord and the use of fetal heart rate auscultation prior to delivery increased significantly, from 17.8% to 99.4% and 35.7% to 83.4%, respectively. Again, the trend for each of these changes reached statistical significance at p<0.05.

**Table 6:** Delivery and selected newborn care characteristics by year in Guatemala, 2010-2013

	2010	2011	2012	2013
Birth attendant, N (%)				

Physician	1,241 (32.1)	1,547 (37.8)	1,851 (45.4)	2,416 (52.9)

Nurse/Midwife/HW	39 (1.0)	96 (2.3)	89 (2.2)	95 (2.1)

TBA	2,575 (66.6)	2,433 (59.5)	2,121 (52.1)	2,028 (44.4)

Family/Other	14 (0.4)	12 (0.3)	12 (0.3)	31 (0.7)

Delivery location, N (%)				

Hospital	1,162 (30.0)	1,570 (38.4)	1,814 (44.5)	2,297 (50.3)

Clinic	104 (2.7)	56 (1.4)	121 (3.0)	215 (4.7)

Home/Other	2,603 (67.3)	2,462 (60.2)	2,138 (52.5)	2,058 (45.0)

Delivery mode, N (%)				

Vaginal	3,350 (86.6)	3,402 (83.2)	3,251 (79.8)	3,461 (75.7)

Vaginal assisted	4 (0.1)	4 (0.1)	12 (0.3)	0 (0.0)

C-section	515 (13.3)	682 (16.7)	809 (19.9)	1,109 (24.3)

Maternal antibiotics, N (%)	405 (12.6)	675 (17.0)	950 (24.2)	1,373 (30.7)

Oxytocics, N (%)	602 (18.7)	1,014 (25.5)	1,155 (29.5)	1,779 (40.0)

Blood transfusion, N (%)	6 (0.2)	9 (0.2)	11 (0.3)	29 (0.7)

Clean razor				

Fetal heart rate taken, N (%)	1,381 (35.7)	1,933 (47.3)	3,340 (82.0)	3,811 (83.4)

BA gloves used, N (%)	3,750 (98.4)	4,043 (99.3)	4,011 (99.2)	4,503 (99.2)

Bag and mask resuscitation, N (%)	28 (0.7)	23 (0.6)	29 (0.7)	76 (1.7)

Breastfeed <1 hr after delivery, N (%)	2,729 (83.6)	3,346 (81.9)	3,200 (78.5)	3,072 (67.1)

As shown in Table 7, the largest decreases in mortality rates occurred among facility deliveries. Neonatal mortality significantly decreased from 38.2 to 20.6 deaths per 1000 live births, stillbirths decreased from 29.0 to 17.7 and perinatal mortality 56.8 to 33.3 per 1,000 births (p<0.05 for trends of reduction in facilities). Although smaller in magnitude and not statistically significant, perinatal mortality and stillbirths also decreased in babies born in the community over this time period from 36.5 to 28.7 and 17.6 to 14.5 per 1000 births, respectively. Neonatal mortality showed a smaller decrease (23.9 to 22.7 per 1,000 births).

**Table 7:** Neonatal, stillbirth and perinatal mortality rates by delivery location and year in Guatemala

	2010	2011	2012	2013	2010-2013 trend, P-value
**Facility mortality rates**					

Facility births, N	1,277	1,643	1,950	2,537	

Neonatal mortality < 28 days, n/N (rate/1000)	47/1,230 (38.2)	34/1,599 (21.3)	26/1,916 (13.6)	51/2,480 (20.6)	0.0001

Stillbirths, n/N (rate/1000)	37/1,277 (29.0)	38/1,643 (23.1)	34/1,950 (17.4)	45/2,537 (17.7)	<0.0001

Perinatal mortality, n/N (rate/1000)	72/1,267 (56.8)	60/1,637 (36.7)	53/1,950 (27.2)	84/2,525 (33.3)	<0.0001

**Community mortality rates**					

Community births, N	2,614	2,474	2,144	2,063	

Neonatal mortality <28 days, n/N (rate/1000)	61/2,557 (23.9)	43/2,431 (17.7)	43/2,110 (20.4)	46/2,029 (22.7)	0.8118

Stillbirths, n/N (rate/1000)	46/2,614 (17.6)	38/2,474 (15.4)	31/2,144 (14.5)	30/2,063 (14.5)	0.3790

Perinatal mortality, n/N (rate/1000)	95/2,603 (36.5)	68/2,469 (27.5)	60/2,141 (28.0)	59/2,059 (28.7)	0.1599

We also stratified our analyses by birth weight to attempt to determine whether changes varied in neonatal mortality and stillbirth rates from 2010 to 2013 by birth weight group. The largest decrease in neonatal mortality occurred in the 1500 – 2499 gram birth weight group with mortality at 28 days decreasing 52%, from 92.4 to 44.4 deaths per 1000 live births over the 4 year period (p=0.0424 for trend). A smaller 22.5% decrease in mortality occurred in ≥ 2500 gram infants, from 17.3 to 13.4 deaths per 1000 live births (p = 0.4510). No consistent changes in neonatal mortality were observed in 1000 to 1499 gram infants (p =0.5767). Stillbirths weighing 1000-1499 grams declined from 400 to 262 per 1000 births (p=0.0591), and in the ≥2500 gram group decreased by 16%, from 45 to 37.7 deaths per 1000 births (p=0.4510).

## Discussion

Evidence from high income countries suggests that the most effective interventions in improving health outcomes of fetuses, newborns and mothers result from upgrades in systems of perinatal care. These have translated into better medical care provided by more and better trained physicians and other obstetric providers during the antenatal and delivery periods, availability of caesarean sections, and neonatal care (including neonatal resuscitation), and of integrated programs that focus on improving pregnancy outcomes [7]. It has been suggested that in LMIC, child survival strategies should direct resources and focus on neonatal health [16]. Improved levels of maternal education have also been found to have a large effect on reduction of child mortality [5]. MDG 4 for Guatemala, which aimed for the reduction of under-five mortality by two thirds, is close to being achieved [16]. Neonatal mortality in Guatemala in 1987 was 36 per 1,000 live births. In 2009, it had reduced to 18 per 1,000 live births [16]. There is a dearth of data on stillbirths in Guatemala, as is the case in many other LMIC [17].

From 2010 to 2013 in the Chimaltenango area of Guatemala, decreases were observed in perinatal mortality, stillbirths and 28 day neonatal mortality. We explored factors that could be related to these decreases by examining changes in the characteristics of mothers, of infants and of health services over this time period. Small changes occurred in the parity and educational level of mothers over time; no other changes were observed in this group.

A large increase occurred in the proportion of births taking place in a hospital or clinic during this time period. The Guatemalan National Congress approved the National Law for a Healthy Maternity and the National Plan to Promote Maternal and Neonatal Health during 2010. Together, the law and corresponding policy change increased access to health care for women and infants during the prenatal period, delivery and pospartum period, and developed surveillance and financing mechanisms. These two laws impacted multiple governmental institutions (ministry of health, municipalities, firefighters) in Chimaltenango, who promoted hospital deliveries and provided transportation, when needed for maternal or neonatal care. We believe that even though women were not given financial incentives for delivering in the hospital, these larger contextual factors created an increase in hospital deliveries.

The overall quality of care in terms of qualified health personnel, and the availability of the components of comprehensive obstetric care improved over this time period. Obstetrics and pediatrics residency programs began early 2010 and 2011, respectively. A new obstetrical wing in the Chimaltenango Hospital opened during January of 2011, and the neonatal intensive care unit during October of 2012. While we cannot isolate the impact of these programs, we believe the increase in skilled attendants as well as the greater attention to quality that often occurs in teaching programs contributed to the improvements noted.

The results also suggested that the quality of antenatal care as measured by the degree of testing and use of iron and vitamins for all births improved. Cesarean section rates in the study population increased from 13.3% to 24.3% over the 4 years and may have contributed to some of the improvements noted. Babies with birth weights between 1500 and 2499 grams appeared to have the largest reductions in mortality rates, which is consistent with the improved (but not specialized) level of care provided at the Chimaltenango hospital. The largest improvements in mortality rates by place of delivery occurred in babies born in the hospital. The deliveries attended by TBAs did not experience the same decrease in mortality over time as the hospital births. These data suggest that consideration should be given to a policy of phasing out home births with TBAs.

We could not disentangle all the reasons for the decline in mortality which seemed in large part related to increasing use of hospitalization for delivery, increasing cesarean section, fewer TBA deliveries and increased physician deliveries, in addition to the instalation of an NICU and residency programs. We believe that this constellation of interventions along with improvements in prenatal care quality together accounted for the reductions in mortality noted. National changes in clinical care guidelines also explain some of these changes, such as the prophylactic use of antibiotics for all caesarean sections, and of oxytocics for the active management of the third stage of labor.

We observed an increase in the proportion of preterm and low birth weight infants over time. We do not know if this increase actually occurred or was the result of better case ascertainment, related in part to the increase in hospital deliveries. It is notable that the decreases in 28 day neonatal mortality, stillbirths and perinatal mortality occurred despite these increases in the reported preterm and low birth weight babies. Because these babies were at higher risk of mortality, this suggests that the reductions in mortality may have been even greater than our results indicate. Changes in gestational age dating methods, such as increased use of ultrasound, may explain part of the observed increase noted in preterm birth, but would not explain the increase in low-birth weight babies that was observed.

This study had a number of potential limitations. First, although every attempt was made to enrol all pregnant women living in the geographic area, in some of the remote areas, especially early in the study, some deliveries may have been missed. Certain types of data like the maternal BMI were based on data collected during pregnancy and not on reported pre-pregnancy weight and height (as is done in many other studies).

## Conclusions

In summary, in a relatively short time period, in the Chimaltenango area of Guatemala which has a history of poor pregnancy outcomes, there were significant improvements in neonatal, fetal and perinatal mortality. These changes were temporally related to major increases in the use of hospitals and skilled birth attendants for delivery, as well as improvements in the quality of delivery care, neonatal care, and prenatal care. Improvements in outcome were greater in facility births and highest in babies weighing 1500 to 2499 grams. It is likely that a smaller proportion of the improvements in outcomes were also due to improvements in maternal educational level and decreased parity of the pregnant population. These observations suggest that rapid improvements in pregnancy outcomes are possible when there is a concerted effort by the Ministry of Health and the medical establishment to improve the health of pregnant women and their newborns.

## Competing interests

The authors declare they have no competing interests.

## Authors' contributions

AG conceived of the concept, with input from RLG and EMM and wrote the initial draft. AG, LF and AML oversaw the study implementation and participated in study monitoring with NFK and KMH. JLM performed the statistical analyses with input from RLG, AG and EMM. All authors reviewed and approved the final manuscript.

## Peer review

Reviewer reports for this article can be found in Additional file 1.

## Supplementary Material

Additional file 1 Click here for file

## Declarations

This article has been published as part of *Reproductive Health* Volume 12 Supplement 2, 2015: Research reports from the NICHD Global Network for Women’s and Children’s Health Research Maternal and Newborn Health Registry. The full contents of the supplement are available online at http://www.reproductive-health-journal.com/supplements/12/S2. Publication of this supplement was supported by grants from the Eunice Kennedy Shriver National Institute of Child Health and Human Development to RTI International.

## References

[B1] Oestergaard ZM Inoue M Yoshida S Retno Mahanani W Gore FM Cousens S Neonatal Mortality Levels for 193 Countries in 2009 with Trends Since 1990: A Systematic Analysis of Progress, Projections, and Priorities PLoS Medicine 2011 8 8 e1001080 10.1371/journal.pmed.1001080 21918640 PMC3168874

[B2] McClure EM Pasha O Goudar SS Chomba E Garces A Tshefu A Goldenberg RL Epidemiology of stillbirth in low - middle income countries: A Global Network Study Acta Obstetricia Gynecology Scandanivia 2011 90 12 1379 1385 10.1111/j.1600-0412.2011.01275.x PMC3412613 21916854

[B3] Lawn JE Cousens S Zupan J 4 million neonatal deaths: When? Where? Why? Lancet 2005 365 9462 9 18 10.1016/S0140-6736(05)71048-5 15752534

[B4] Blencowe H Cousens S Chou D Oestergaard M Say L Moller A Born Too Soon: The global epidemiology of 15 million preterm births Reproductive Health 2013 10 Suppl 1 S2 10.1186/1742-4755-10-S1-S2 24625129 PMC3828585

[B5] Wang H Liddell CA Coates M Global, regional and national levels of neonatal, infant, and under - 5 mortality during 1990 - 2013: a systematic analysis for the Global Burden of Disease Study 2013 Lancet 2014 384 9947 957 979 10.1016/S0140-6736(14)60497-9 24797572 PMC4165626

[B6] Oza S Cousens S Lawn JE Estimation of daily risk of neonatal death, including the day of birth, in 186 countries in 2013: a vital registration and modelling - based study Lancet Global Health 2014 2 11 e635 e644 10.1016/S2214-109X(14)70309-2 25442688

[B7] Goldenberg RL McClure EM Maternal, fetal and neonatal mortality: lessons learned from historical changes in high income countries and their potential application to low-income countries Maternal Health, Neonatology and Perinatology 2015 1 3 10.1186/s40748-014-0004-z PMC4772754 27057321

[B8] Saleem S McClure E Goudar SS Patel A Esamai F Garces A Goldenberg RL A prospective study of maternal, fetal and neonatal deaths in low - and middle-income countries Bulletin of the World Health Organization 2014 92 8 545 620 10.2471/BLT.13.127464 PMC4147405 25177075

[B9] Knoll Rajaranatnam J Marcus JR Flaxman AD Wang H Levin-Rector A Dwyer L Neonatal, postneonatal, childhood, and under -5 mortality for 187 countries, 1970 - 2010: a systematic analysis of progress towards Millennium Development Goal 4 Lancet 2010 375 9730 1988 2008 10.1016/S0140-6736(10)60703-9 20546887

[B10] Lawn J Shibuya K Stein C No cry at birth: global estimates of intrapartum stillbirths and intrapartum - related neonatal deaths Bulletin of the World Health Organization 2005 83 6 409 417 15976891 PMC2626256

[B11] McClure EM Goldenberg RL Bann CM Maternal mortality, stillbirth and measures of obstetric care in developing and developed countries International Journal Gynec Obstetrics 2007 96 2 139 146 10.1016/j.ijgo.2006.10.010 17274999

[B12] Goldenberg RL McClure EM Bhutta ZA Belizan JM Reddy UM Rubens CE Stillbirths: the vision for 2020 Lancet 2011 377 9779 1798 1805 10.1016/S0140-6736(10)62235-0 21496912

[B13] Mason E McDougall L Lawn JE Gupta A Claeson M Pillay Y From evidence to action to deliver a healthy start for the next generation Lancet 2014 384 9941 455 467 10.1016/S0140-6736(14)60750-9 24853599

[B14] Garces A Mcclure EM Chomba E Patel A Pasha O Tshefu A Goldenberg RL Home birth attendants in low income countries: who are they and what do they do? BMC Pregnancy and Childbirth 2012 12 34 1 9 22583622 10.1186/1471-2393-12-34 PMC3493311

[B15] United Nations Development Program Cifras para el Desarrollo Humano Chimaltenango 2015 Obtained from http://desarrollohumano.org.gt/sites/default/files/04%20Fasciculo%20Chimaltenango.pdf (accessed April 16, )

[B16] United Nations Development Program Millenium Development Goals 2014 Obtained from http://mdgs.un.org/unsd/mdg/Resources/Static/Products/Progress/Snapshots/GTM.pdf (accessed February 5, ero de 2015)

[B17] Ministry of Public Health and Social Assistance of Guatemala National Statistics Institute 2008 - 2009 National Maternal Infant Health Survey Guatemala Ministry of Public Health 2011

