# Supplementary material for: Trends in perinatal deaths from 2010 to 2013 in the Guatemalan Western Highlands
Source: Reprod Health. 2015 Jun 8;12(Suppl 2):S14. doi: 10.1186/1742-4755-12-S2-S14 (PMC4464607; doi:10.1186/1742-4755-12-S2-S14)
Supplement: Additional file 1 [file 1742-4755-12-S2-S14-S1.pdf]

Referee's comments to the authors– this sheet WILL be seen by the author(s) and published with the article

Title	Trends in perinatal deaths from 2010 to 2013 in the Guatemalan Western Highlands
Author(s)	Ana Garces, Elizabeth M Mcclure, K Michael Hambidge, Nancy F Krebs, Lester Figueroa, Marta Lidia Aguilar, Janet L Moore, Robert L Goldenberg
Referee's name	Bob Silver

When assessing the work, please consider the following points, where applicable:

1. Is the question posed by the authors new and well defined?
2. Are the methods appropriate and well described, and are sufficient details provided to replicate the work?
3. Are the data sound and well controlled?
4. Does the manuscript adhere to the relevant standards for reporting and data deposition?
5. Are the discussion and conclusions well balanced and adequately supported by the data?
6. Do the title and abstract accurately convey what has been found?
7. Is the writing acceptable?

Please make your report as constructive and detailed as possible in your comments so that authors have the opportunity to overcome any serious deficiencies that you find and please also divide your comments into the following categories:

- Major Compulsory Revisions (which the author must respond to before a decision on publication can be reached)
- Minor Essential Revisions (such as missing labels on figures, or the wrong use of a term, which the author can be trusted to correct)
- Discretionary Revisions (which are recommendations for improvement but which the author can choose to ignore)

Where possible please supply references to substantiate your comments.

When referring to the manuscript please provide specific page and paragraph citations where appropriate.

General comments:

This is an original study describing changes over time in perinatal deaths in a rural area of Guatemala after implementation of a program to increase in hospital deliveries with skilled birth attendants. The authors report significant reductions in perinatal mortality and neonatal mortality during the study interval. The data are extremely important and of interest to readers of the journal. Initiation of similar programs has the potential to improve perinatal outcomes in other similar areas.

Major compulsory revisions:

Minor essential revisions:

1. The objective or purpose of the study should be more clearly stated.
2. The intervention should be more clearly described with increased detail.
3. Did the authors do anything in an attempt to assess how complete their ascertainment was? Are there any community or National records that could be used as a "gold standard?"
4. Further analysis in attempt to assess which factors contributed to improved outcomes and to control for potential confounding should be performed. The current analysis is quite descriptive.

Referee's comments to the authors– this sheet WILL be seen by the author(s) and published with the article

Title	Trends in perinatal deaths from 2010 to 2013 in the Guatemalan Western Highlands
Author(s)	Ana Garces, Elizabeth M Mcclure, K Michael Hambidge, Nancy F Krebs, Lester Figueroa, Marta Lidia Aguilar, Janet L Moore, Robert L Goldenberg
Referee's name	Elizabeth Stringer

Title of paper: Trends in perinatal deaths from 2012 to 2013 in the Guatemalan Western Highlands

Overview:

This is a secondary analysis of the Global Network Study that describes perinatal mortality rates, stillbirth rates, and maternal mortality rates over a four-year period. The paper is exciting because it shows a rather dramatic decline in the perinatal mortality rates in the described period, but the reader is left wondering the reasons for this decline which are not well described.

Introduction

The introduction is clear and well written. At the end of paragraph one the authors state, "there is evidence of accelerating declines in neonatal mortality from 2000 to 2010 in many LMIC". I would like the authors to elaborate on reasons for some of these declines. In paragraph 3, the authors state the "TBAs in the region". Please describe which region. In the last paragraph of the introduction, the authors switch verb tense. Please make consistent.

Methods

Please add in specific dates for the start and stop of this study. Is there a specific reason the authors chose this timeframe?

More description is needed regarding the ten clusters and where deliveries occur. In many low and middle income countries, deliveries occur in health centers, hospitals, and at home. In this area, it seems that the majority of deliveries occur either in a hospital or a home. Can the authors please elaborate on how the health centers are situated in the different clusters. How many there are and how many hospitals there are? Can the authors please describe any referral systems in place? Additionally, what proportion of the deliveries in the entire Chimaltenango region over the time period were in those ten clusters? A map of these clusters and where the health centers and hospitals are located could be helpful.

Are the auxiliary nurses, study nurses? If so, please clarify.

How were pregnancies diagnosed? Complications from first trimester pregnancies are a major contributor to maternal mortalities. Were these included in your study? Perinatal mortality rates were defined as fetal deaths >20 weeks, but the authors do not describe how the estimated gestational age of a pregnancy was determined. Did the study routinely provide ultrasounds? How was preterm birth defined?

Please include on page 5, how the different rates were adjusted for by cluster.

Results

This is a prospective cohort study, however, we are not provided the initial numbers of women enrolled in the prenatal period and how many were lost to follow up. One suggestion would be to provide a flow chart with the numbers of women enrolled and the numbers of women with deliveries and then the number of neonatal outcomes.

How were twin births handled in the analysis?

There are no statistical tests for trend on Tables 2-6. Can the authors please provide at least p values for trend.

In the paragraph on quality of care characteristics, the authors state that the use of antibiotics increased. Can the authors please elaborate on the indications on why these antibiotics were given. An increase in antibiotic usage is not necessarily a good thing unless they are given appropriately.

Discussion

In the second paragraph of the discussion, the authors state that "they explored factors that could be related to declined in rates of stillbirth and perinatal mortality rates" but the reader is left not knowing why the declines occurred. Could the authors please

provide more detail on the increase in births occurring in the hospital or clinic(s)? Were there government policies that increased the number of facility births? Were there incentives given to women? Did studies facilitate women delivering in health facilities?

The authors mention that a residency program started as well as a NICU. Please provide more detail on the exact timing of this and the impact it could have had.

Finally, the authors fail to comment on the high cesarean delivery rate of 25%. WHO recommends a cesarean delivery rate around 15%. Could this be the sole driver of the decline in perinatal mortality rates?

Supplement Editor's comments:

Please put in the background section of the abstract the objective of your study. Some description about the characteristics of the Chimaltenango area would be of interest jointly with peculiarities of this region in comparison with the country.
